# Proteomic analysis predicts anti-angiogenic resistance in recurred glioblastoma

**DOI:** 10.1186/s12967-023-03936-8

**Published:** 2023-02-02

**Authors:** Hanwool Jeon, Joonho Byun, Hayeong Kang, Kyunggon Kim, Eunyeup Lee, Jeong Hoon Kim, Chang Ki Hong, Sang Woo Song, Young-Hoon Kim, Sangjoon Chong, Jae Hyun Kim, Soo Jeong Nam, Ji Eun Park, Seungjoo Lee

**Affiliations:** 1 Translational Biomedical Research Group, Asan Institute for Life Sciences, Asan Medical Center, Seoul, Republic of Korea; 2 Department of Neurological Surgery, Brain Tumor Center, Asan Medical Center, University of Ulsan College of Medicine 88, Olympic-ro 43-gil, Songpa-gu, Seoul, Republic of Korea; 3 Asan Institute for Life Sciences, Asan Medical Center, Seoul, Republic of Korea; 4 Department of Pathology, Asan Medical Center, University of Ulsan College of Medicine, Seoul, Republic of Korea; 5 Department of Radiology and Research Institute of Radiology, Asan Medical Center, University of Ulsan College of Medicine, Seoul, Republic of Korea; 6 Bio-Medical Institute of Technology, University of Ulsan College of Medicine, Seoul, Republic of Korea

**Keywords:** Anti-angiogenic resistance, Prediction biomarker, Proteomics

## Abstract

**Background:**

Recurrence is common in glioblastoma multiforme (GBM) because of the infiltrative, residual cells in the tumor margin. Standard therapy for GBM consists of surgical resection followed by chemotherapy and radiotherapy, but the median survival of GBM patients remains poor (~ 1.5 years). For recurrent GBM, anti-angiogenic treatment is one of the common treatment approaches. However, current anti-angiogenic treatment modalities are not satisfactory because of the resistance to anti-angiogenic agents in some patients. Therefore, we sought to identify novel prognostic biomarkers that can predict the therapeutic response to anti-angiogenic agents in patients with recurrent glioblastoma.

**Methods:**

We selected patients with recurrent GBM who were treated with anti-angiogenic agents and classified them into responders and non-responders to anti-angiogenic therapy. Then, we performed proteomic analysis using liquid-chromatography mass spectrometry (LC–MS) with formalin-fixed paraffin-embedded (FFPE) tissues obtained from surgical specimens. We conducted a gene-ontology (GO) analysis based on protein abundance in the responder and non-responder groups. Based on the LC–MS and GO analysis results, we identified potential predictive biomarkers for anti-angiogenic therapy and validated them in recurrent glioblastoma patients.

**Results:**

In the mass spectrometry-based approach, 4957 unique proteins were quantified with high confidence across clinical parameters. Unsupervised clustering analysis highlighted distinct proteomic patterns (n = 269 proteins) between responders and non-responders. The GO term enrichment analysis revealed a cluster of genes related to immune cell-related pathways (e.g., TMEM173, FADD, CD99) in the responder group, whereas the non-responder group had a high expression of genes related to nuclear replisome (POLD) and damaged DNA binding (ERCC2). Immunohistochemistry of these biomarkers showed that the expression levels of TMEM173 and FADD were significantly associated with the overall survival and progression-free survival of patients with recurrent GBM.

**Conclusions:**

The candidate biomarkers identified in our protein analysis may be useful for predicting the clinical response to anti-angiogenic agents in patients with recurred GBM.

**Supplementary Information:**

The online version contains supplementary material available at 10.1186/s12967-023-03936-8.

## Background

Glioblastoma multiforme (GBM) is one of the most aggressive cancers with only a 1.5-year overall survival duration despite the availability of multiple treatment options. Angiogenesis is a common feature of the tumor microenvironment of GBM, which provides energy for tumor migration and development. Angiogenic factors such as VEGF (vascular endothelial growth factor), FGF-2 (fibroblast growth factor 2) [1], PDGF (platelet-derived growth factor) [2], angiopoietins [3], and ephrines [4] induce neovascularization around the tumor. Bevacizumab, a humanized monoclonal antibody that inhibits the VEGF-mediated signaling pathway, is a potent anti-angiogenic drug for treating recurred GBM. Several studies showed that while bevacizumab extends progression-free survival and improves the quality of life in GBM patients, it is less effective in improving overall survival [5–7]. Additionally, this monoclonal antibody is used to treat various types of cancer, including lung cancer [8], colon cancer [9], breast cancer [10], ovarian cancer [11], renal cell carcinoma [12], colorectal cancer [13], and cervical cancer [14]. However, anti-angiogenic agents decrease tumor perfusion and oxygenation, and induce acidosis. Paradoxically, these biological consequences could enhance the VEGF signalling pathway via the upregulation of the hypoxic-inducible factor-1 (HIF-1)-α.

Resistance to anti-angiogenic therapy is mediated by the recruitment of vascular endothelial progenitor cells [15], tumor invasion and migration, cancer stem cell adaptation [16], and tumor cell dormancy [17]. While biomarkers used for the diagnosis of GBM, such as TERT (telomerase reverse transcriptase) [18], MGMT (O-6-Methylguanine-DNA Methyltransferase) [19], CD44 [20], ATRX (alpha-thalassemia/mental retardation, X-linked) [21], MMP9 (matrix metallopeptidase 9) [22], TNF-alpha (tumor necrosis factor-alpha) [23], S100A8 (S100 Calcium Binding Protein A8) [24], MCT1 (Monocarboxylate transporter 1) [25], and thrombospondin-1[26] can predict the prognosis in glioblastoma patients, it is difficult to predict the clinical outcome after anti-angiogenic treatment using those biomarkers. Accordingly, the discovery of biomarkers that can predict the susceptibility of anti-angiogenic agents in individual patients would significantly improve the efficacy of treatment and reduce side effects.

For predicting the response to anti-angiogenic treatment, biomarkers can be directly analyzed in tumor tissues at the gene and protein levels, while non-invasive imaging techniques such as magnetic resonance imaging (MRI) and computed tomography (CT) have also shown predictive potentials. There are two types of resistance after anti-angiogenic treatment: adaptive resistance and intrinsic resistance [27]. Adaptive resistance is related to increases in pro-angiogenic factors [28, 29], vascular progenitor cells from the bone marrow [30], and local stromal cells (e.g., pericytes) around the tumor [31]. Intrinsic resistance is another mechanism of resistance, which involves difficulty in inhibiting the tumor target signaling because of the secretion of pro-angiogenic factors by immune cells surrounding the tumors. This phenomenon can be observed by detecting increases in pro-angiogenic factor levels through pathologic analysis or via enhanced MRI. However, because these methods were predominantly performed in preclinical research, whether they can sufficiently describe the actual tumor environment is unknown.

Analysis of resistance mechanisms has been performed using single-nucleotide polymorphisms [32], miRNAs[33], proteomics [34] or exosomes [35], quantifying microvascular density in FFPE tissues, estimating interstitial fluid pressure [36], and confirming oxygen tension [37]. These methods showed inconsistent results because the tumor tissues were collected from the different parts of the tumor, making it difficult to establish a standart protocol for specimen preparation. Patient samples such as blood and urine require minimal invasion but are disadvantaged in showing variable results depending on the patient's health status.

In this study, we performed a TMA-based proteomic analysis on tumor cores that were obtained from surgical specimens. This method has the advantage of concurrently analyzing multiple tumor tissues with a minimal amount of tissue samples. By combining LC mass spectroscopy data, we attempted to identify the biomarkers that can predict the response to anti-angiogenic treatment in GBM patients.

## Methods

### Study design

Of the patients with recurrent GBM (WHO grade IV) who received anti-angiogenic therapy at Asan Medical Center (Seoul, Republic of Korea), those meeting the following inclusion criteria were selected for this study: (1) diagnosis of GBM based on pathology, (2) aged 19–80 years, (3) treated with concurrent chemoradiotherapy using temozolomide (Stupp protocol), and (4) had available follow-up MRI including pre-contrast and contrast-enhanced T1-weighted imaging (CE-T1W1). We excluded those with (1) indistinguishable recurrent (non-target lesion) and necrosis after radiotherapy, (2) low Karnofsky Performance Scale score (< 40), or (3) very small tissue specimens. This retrospective study was approved by the institutional review board of Asan Medical Center (IRB no. 2016–1245, 2017–0665, 2019–0082).

The standard concurrent chemoradiation therapy (CCRT) procedure [38] used at our center was fractionated focal radiotherapy at a dose of 2 Gy per fraction, given once a day for five days a week for six weeks to reach a total dose of 60 Gy. The standard CCRT also used temozolomide at a dose of 75 mg per m^2^ per day, given seven days a week from the first day of radiotherapy to the last day of radiotherapy. Prior to a four-week break, the patients had received up to 6 cycles of adjuvant temozolomide every four weeks on a five-day schedule. The first dose was 150 mg per m^2^, and the dose was increased to 200 mg per m^2^ for the second cycle if there were no side effects.

Patients were deemed to have recurrence when they had a new or increasing (> 25%) measurable contrast-enhanced mass greater than 1 × 1 cm at a scan obtained 12 weeks after standard CCRT or later. At the end of treatment break, pseudo-progression was ruled out in strict accordance with the previously published protocol [39]. Bevacizumab (Avastin; 10 mg/kg; Roche) or temozolomide (Temodal; 150 mg/day for five days every 28 days; MSD) were used as second-line treatments for patients with recurrence.

### Discovery cohort

Seven patients with a very favorable prognosis and seven patients with unfavorable prognosis due to rapid recurrent and limited survival were selected for protein analysis. For biomarker discovery, we identified 163 patients who were treated with bevacizumab for the recurrent GBM between 2010 and 2016 at our center; of them, we excluded 71 patients because the patients were treated with the partial resection or stereotactic biopsy or follow-up loss. Among 92 patients, the 20 patients were also excluded because whose specimens were not passed quality control (QC) test for the proteomic analysis(Table 1). The residual 72 patients were ranked based on survival duration by descending order. The 7 patients with upper survival duration (responder group) and the 7 patients with lower survival duration (non-responder group) were selected after propensity score matching test. There were no differences in the baseline clinical characterisitics of the responder and non-responder group except the survival duration after bevacizumab treatment. (Table 2-clinical characteristics) Responsiveness to treatment was determined retrospectively by selecting patients who were present at both ends and calculating the time interval between anti-angiogenic treatment and recurrence.

**Table 1:**
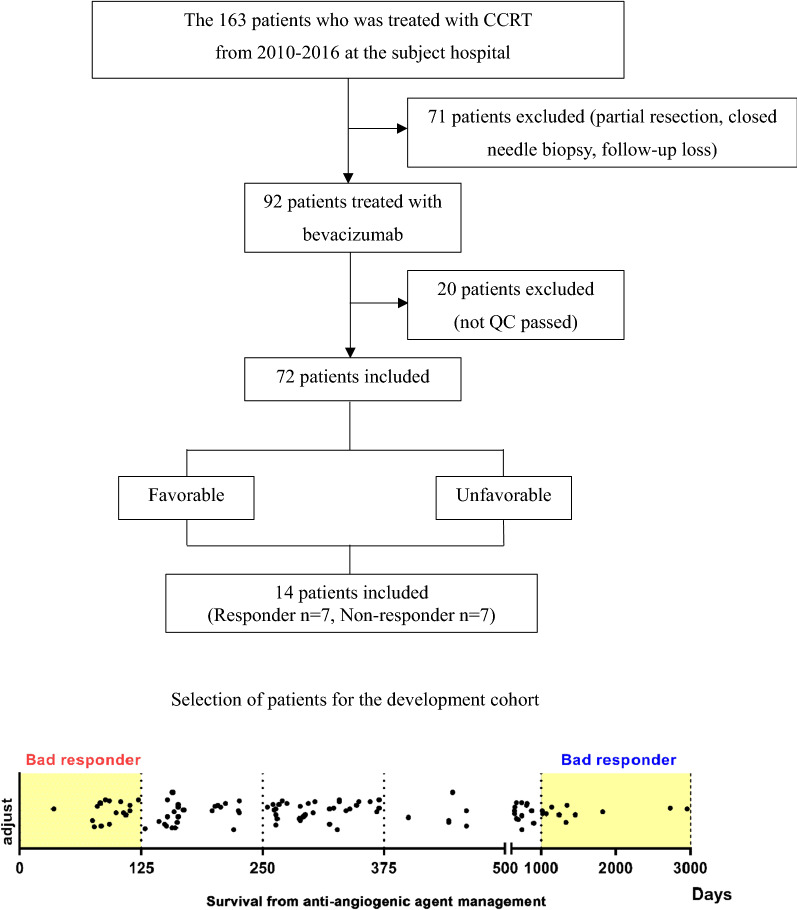
Patient cohort (biomarker development cohort)

**Table 2:** Demographics of GBM patients for marker development >

Characteristics	Responder (n = 7)	Non-responder (n = 7)	P-value
Age	56.71 ± 6.767	57.43 ± 2.224	0.9218
Gender	Male (n = 6)	Male (n = 2)	
	Female (n = 1)	Female (n = 5)	
Molecular type			
IDH wild type	3	3	
MGMT promoter status (methylated/unmethylated/NA)	0/4/3	0/1/6	
Surgical resection type			
Partial resection	1	3	
Gross total resection	6	4	
TMZ duration	354.7 ± 98.07	306.9 ± 89.22	0.7244
Pre-Avastin KPS	60 ± 4.364	50 ± 5.774	0.1922
Overall survival (days)	828.6 ± 91.21	771.1 ± 172.6	0.7736
Progression free survival (days)	277 ± 71.79	458 ± 83.35	0.1258
Avastin dose (mg/kg)	685.7 ± 40.41	595.7 ± 22.24	0.0748
Initial tumor size (mm^3^)	20710 ± 5902	28653 ± 6692	0.3909
Recurred tumor size (mm^3^)	37549 ± 12,339	23238 ± 6398	0.3235
TMZ + AVASTIN	n = 7	n = 7	
Mono therapy	0	0	

### Validation cohort

For the validation cohort, we first identified 223 patients with recurrent glioblastoma who were treated with bevacizumab between 2017 and 2020 at our hospital. Of them, we excluded those with insufficient tissue specimens for histological analysis (n = 101), and those who were treated with bevacizumab for less than 4 weeks (n = 29). Finally, 93 patients were included in the validation cohort (Table 3).

**Table 3:**
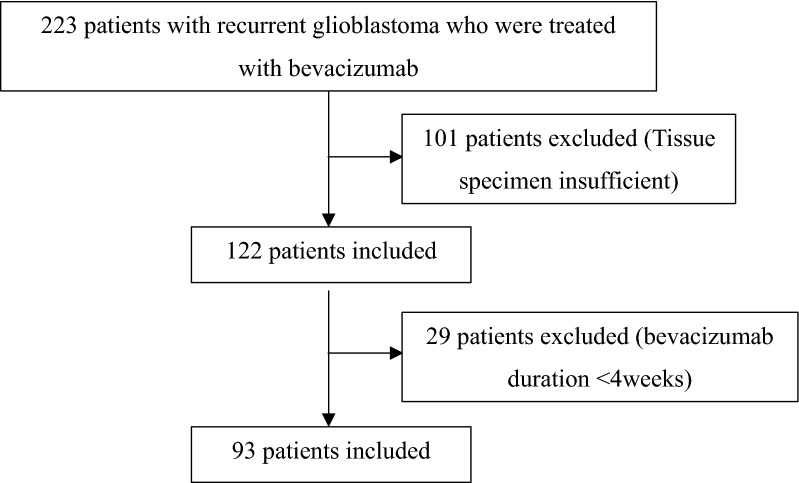
Patient cohort (Validation cohort)

### Response assessment

MRI scans were performed every two to three months. Following a second look operation or a clinico-radiological assessment, a pathologist confirmed tumor progression (S.J.N. with more than 10 years of clinical experience in pathology). Clinico-radiological diagnoses were made by the consensus among three neuro-oncologists (J.H.K., Y.H.K., S.L.) and a radiologist (J.E.P.), all of whom had more than 10 years of clinical experience, according to the Response Assessment in Neuro-Oncology (RANO) criteria [40]. At the time of progression, imaging patterns were determined according to whether the increased contrast enhancement or T2/FLAIR high-intensity signal involved the primary site. The three main types of progression recorded were (1) enhancing local progression (focus of the contrast enhancement at or within 3 cm of the primary site); (2) non-enhancing diffuse progression (stable local contrast-enhancing tumor but an area of abnormal FLAIR hyperintensity is not concordant and extends more than 3 cm from the primary site); and (3) distant progression (new focus of contrast enhancement or an area of abnormal FLAIR hyperintensity extending more than 3 cm from the primary site with intervening normal-appearing white matter). The judgment of the progression pattern was made by a consensus between two neuroradiologists.

Progression-free survival (PFS) was defined as the time from secondary treatment with bevacizumab until the first imaging report indicating worsening/progression (based on the RANO criteria) or death. Overall survival (OS) was defined as the time from secondary treatment with bevacizumab or temozolomide until death.

### Proteomic analysis of GBM tissue samples using mass-spectrometry

To detect proteins in tissue samples, paraffin blocks were sectioned into 10-μm-thick slides. The tissues were collected in a 1.5 mL tube, mixed with 0.5 mL heptane, and incubated at room temperature for 1 h. Then, 25 μl methanol was added, vortexed for 10 s, and centrifuged for 2 min at 9000 × g. After carefully removing the supernatant, the resulting pellet was air-dried for 5 min and vortexed with 100 μl of EXB plus extraction buffer and beta-mercaptoethanol. After 5 min of incubation, the mixture was vortexed and heated for 20 min at 100 °C. Thermomixer was used to incubate the mixture at 80 °C for 2 h at 750 rpm. Then, the sample was cooled for 1 min at 4 °C. The supernatant was transferred to a 1.5 ml tube after centrifugation at 14,000 × g for 15 min at 4 °C.

BCA assay was used to measure the quantity of protein. After melting the protein pellet, 10 μl of 25 mM NH_4_HCO3 (50 mM DTT) was added and incubated in a Thermomixer for 1 h at 37 °C at 950 rpm. Then, 10 μl of 25 mM NH_4_HCO3 (10 mM iodoacetamide) was added and mixed for 1 h at 37 °C at 950 rpm. After then, 90 μl of 25 mM NH_4_HCO3 was added, and 20 μl of buffer with 0.25 μg/μl trypsin was added and digested at 37 °C. Lastly, 20 μl of 5% TFA solution was added to stop the reaction, and the mixture was mixed at 950 rpm for 1 h at 37 °C. The peptide-containing supernatant was transferred to a 0.5 ml tube and vacuum-dried after centrifugation at 13,000 rpm for 30 min at room temperature. Proteins were identified using LC-HRMS technique according to the conditions.

**Table:** 

Liquid chromatography condition	
Column	50 cm length, 75um I.D, 360 um O.D fused silica C18
LC rum time	200 min
Flow rate	350 nl/min
Gradient	5% Sol B to 50% Sol B during 150 min gradient
Sol A	0.1% Formic acid with 5% DMSO
Sol B	80% acetonitrile, 0.1% formic acid with 5% DMSO

**Table:** 

Mass spectrometry	
MS 1 resolution	70000
MS 1 maximum fill time	20 ms
MS 2 resolution	17500
MS 2 maximum fill time	100 ms
Auto gain control	1e6

### Pathology analysis and tissue microarray (TMA) block production

The core regions of tumors were selected by staining the slides using hematoxylin and eosin. Tissue sections were deparaffinized by heating at 60 °C, followed by passages through xylene and alcohol stages. After 3 min of incubation with hematoxylin, the sample was rinsed with deionized water. After dipping the sample in acetic acid and bluing solution, the remaining solution was eliminated with deionized water. After 3 min of eosin staining, the slide was dehydrated in serial incubation in 90% ethanol, 100% ethanol, and xylene, and finally mounted with a permanent mounting solution. Two tumor tissues were punched with a circular size of 2 mm to acquire samples. Blocks were made according to the cohort arrangement of tumor tissue. The TMA blocks were cut into 4-μm sections and used for immunohistochemistry and hematoxylin-and-eosin staining.

### Immunohistochemistry, image processing, and acquisition

Tissue slides were heated for 30 min in a dry oven at 60 °C to dissolve paraffin. De-paraffinization was then performed by dipping the slide three times in xylene for 10 min each time, and serial incubation in decreasing alcohol solutions to eliminate any remaining xylene. The antigen-retrieval process was used to adjust the pH according to each antibody and boiling was performed in a microwave. The tissue slides were incubated with 3% hydrogen peroxide for 10 min to eliminate the production of endogenic peroxidase. For nucleus staining, the tissues were permeabilized using 0.1% TBS-T buffer for 10 min, followed by a 30-min blocking step with 2.5% normal horse serum to decrease non-specific binding. Primary antibodies were diluted in 0.3% TBS-T and incubated overnight. After 24 h, the slides were washed three times for 10 min with 0.1% TBS-T. The antibodies used in immunohistochemistry were anti-TMEM173 (1:5000; Proteintech, Cat#19851–1-AP), anti-FADD (1:500; NOVUS, Cat# NBP1− 81831), anti-CD99 (1:150; ORIGENE, Cat#UM800151), anti-POLD1 (1:500; Proteintech, Cat#15646–1-AP), anti-ERCC2 (1:200; Proteintech, Cat#10818–1-AP). Then, the slides were incubated for 30 min at room temperature with the universal pan-specific (anti-mouse/rabbit/goat IgG) secondary antibody included in universal quick kits (VECTOR laboratories). The secondary antibody washing step was repeated three times for 10 min at room temperature using 0.1% TBS-T. Then, the slides were incubated with a peroxidase streptavidin complex for 10 min. Afterward, the color was developed using a DAB substrate kit and rinsed after 5 min. For nucleus staining, the slides were incubated with hematoxylin for 3 min, then added to alcohol, dipped in xylene, and mounted to observe under a microscope. According to the signal intensity, IHC slides were categorized into negative (no signal), low (weak signal), and high (moderate-to-strong signal).

### Statistical analysis

Statistical analyses were performed using SPSS software (IBM Corp., Armonk, NY, USA). Statistical significance was evaluated in all patients without removing outliers. Statistical analysis using the Kaplan–Meier method were performed in the high-expression and low-expression groups to investigate whether the survival outcomes differed between the two groups. For all analyses, P values < 0.05 were considered statistically significant.

## Results

### Selection of patients with recurrent GBM following anti-angiogenic therapy for proteomic analysis

**Fig. 1:**
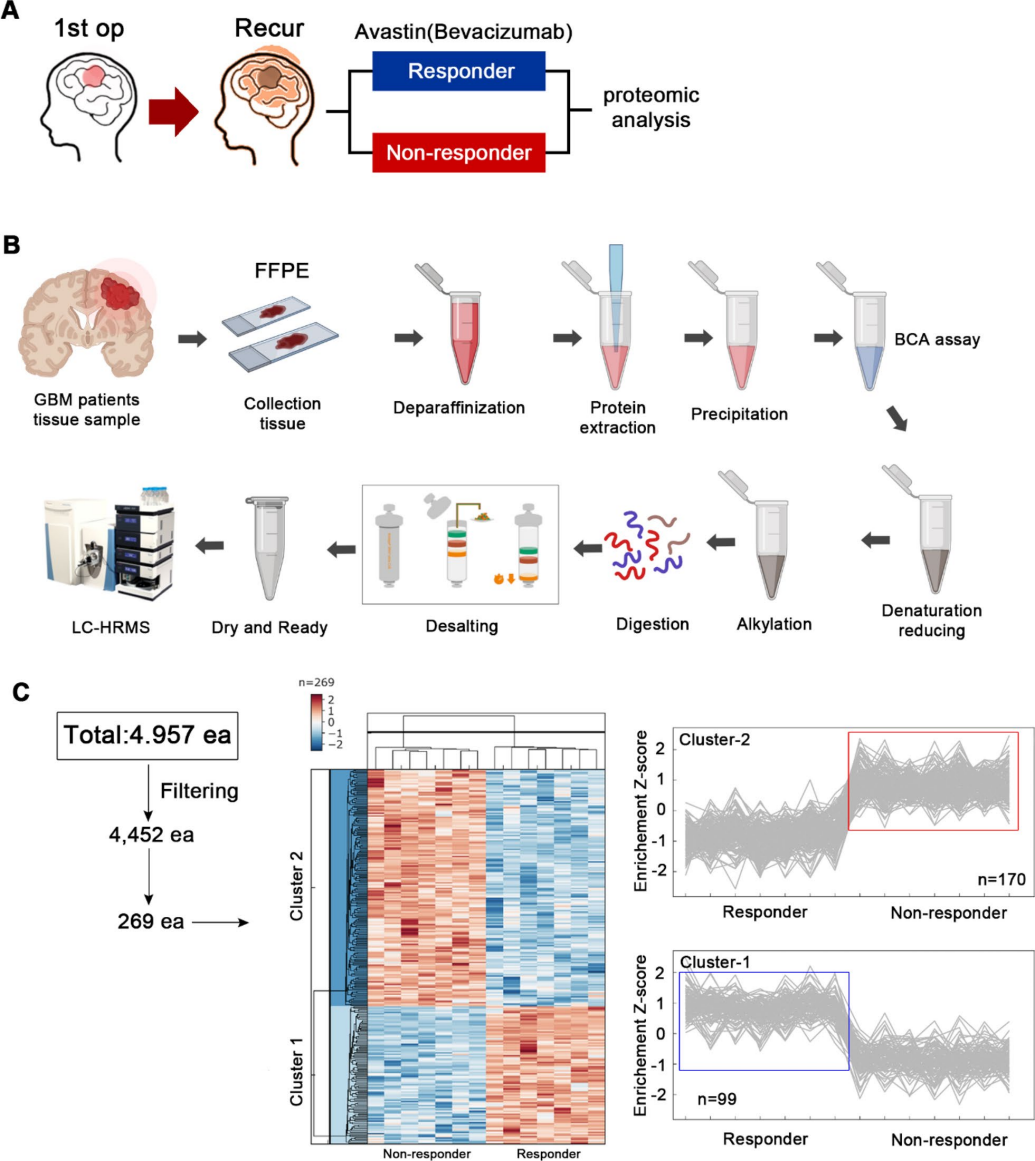
Proteomic profiling of recurred GBM patients. **A** A total of 14 patients with recurred GBM were divided according to their treatment response and included in the proteomic analysis. **B** Schematic diagram of proteomic analysis using liquid chromatography-high resolution mass spectrometry (LC-HRMS) on tumor tissue paraffin slides. **C** Heatmap analysis of 269 proteins with statistical significance from 4957 proteins. Of them, 99 proteins and 170 proteins were highly expressed in the Responder group and the Non-responder group, respectively

We selected a total of 14 patients with recurrent GBM after anti-angiogenic therapy (Responder group, n = 7; Non-responder group, n = 7) to identify protein biomarkers for the responsiveness of anti-angiogenic treatments (Fig. 1A). The characteristics and treatment outcomes of the Responder group and the Non-responder group are shown in Tables 1, 2. The two groups did not show significant differences in age, pre-Avastin Karnofsky Performance Scale [41], molecular type (i.e., IDH status, MGMT promoter status), and surgical resection type (i.e., partial resection vs. gross total resection). The bevacizumab dose was 685.7 mg/kg in the Responder group and 595.7 mg/kg in the Non-responder group (P = 0.075). The initial tumor size was 20,710 mm^3^ and 28,653 mm^3^ in the Responder group and the Non-responder group, respectively (P = 0.39), and the recurred tumor size after standard therapies was 37,549 mm^3^ and 23,238 mm^3^ in the Responder group and the Non-responder group, respectively (P = 0.32). Recurrence was noted after an average of 61 days after bevacizumab treatment in the Non-responder group and after 381 days in the Responder group. Except the responsiveness to Avastin(bevacizumab), all demographic profiles and molecular features of glioblastomas were not statsically different between responder versus non-responder group.

For proteomic analysis, tumor core punches from fixed paraffin tissues were used after pathological analysis. The tumor tissues used for proteomic analysis were obtained from specimens at first operation. Protein isolation was performed using mass spectrometry (Fig. 1B). A total of 4,957 proteins were detected, and Benjamin-Hochberg false discovery rate (FDR) was applied to cluster proteins with significant values in the Responder and Non-responder groups (Fig. 1C). After grouping the proteins according to their Z-scores, 170 proteins were found to be significantly more abundant in the Non-responder group, while 99 proteins were more abundant in the Responder group.

### Cluster identification analysis of recurred GBM patients

**Fig. 2:**
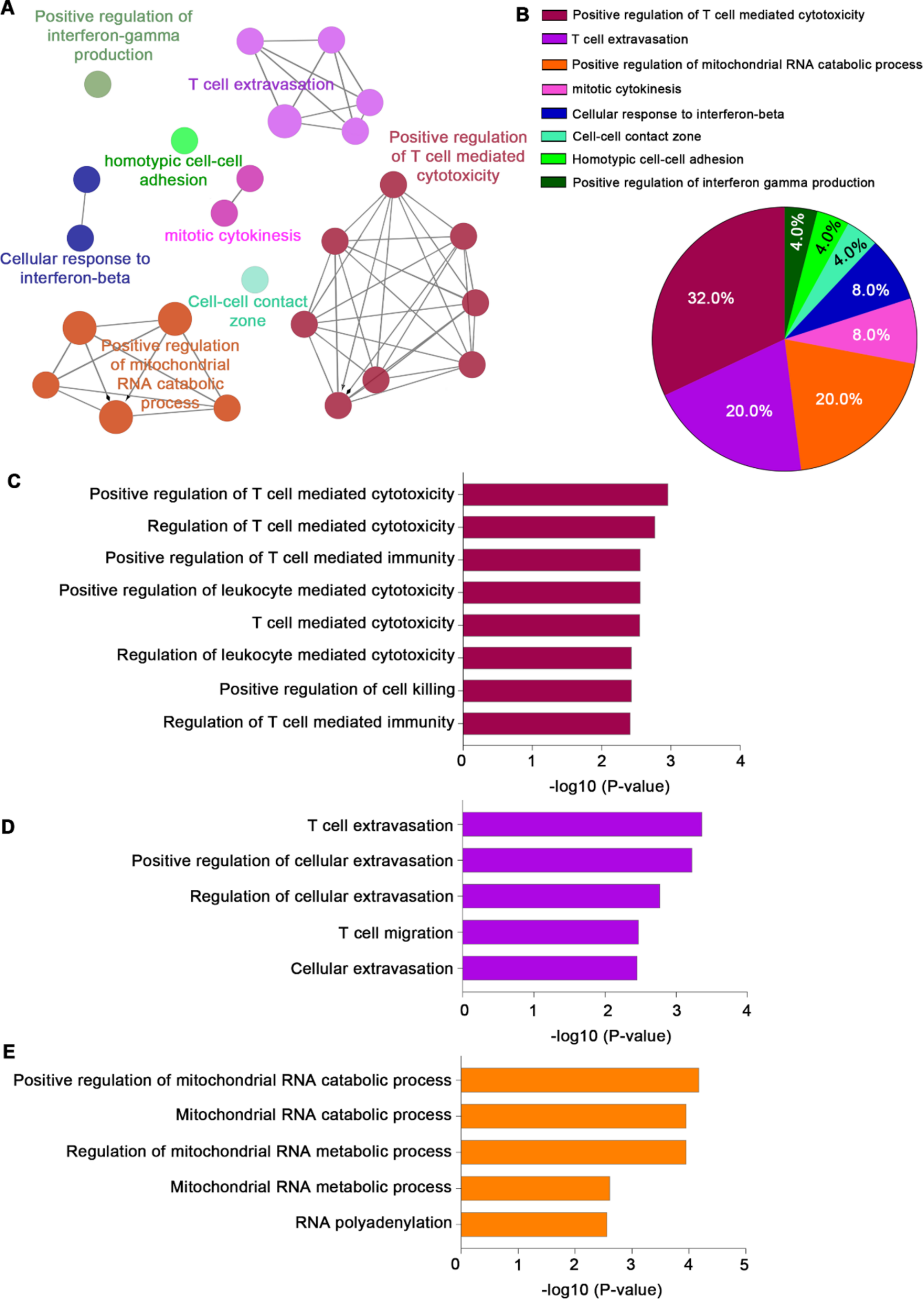
Gene ontology patterns and significant pathways in the Responder group. **A** Cluster analysis results in the Responder group. **B** Pie charts showing the gene ontology classifications. Bar graphs of gene ontology enrichment analysis for pathways related to **C** T cells, **D** T cell extravasation, and **E** RNA catabolic process

The functionality of the identified proteins was verified by assessing the association of each protein. The identified proteins were matched to the gene-ontology (GO) database to determine the pathway for each patient group (Fig. 2A). In the Responder group, various immune-related pathways were identified. T cell extravasation and positive regulation of mitochondrial RNA catabolic processes each accounted for 20% of the total, while positive regulation of T cell-mediated cytotoxicity accounted for 32%. Cellular response to interferon-beta and mitotic cytokinesis accounted 8%. The remaining pathways were associated with cell–cell contact zone, homotypic cell–cell adhesion, positive regulation of interferon-gamma production (Fig. 2B). Various signaling pathways, including the regulation of T cell-mediated cytotoxicity, leukocyte-mediated cytotoxicity and cell killing were included in the positive T cell-mediated cytotoxicity with a proportion of 32% (Fig. 2C). Cellular extravasation and T cell migration were included in the 20% T cell extravasation pathway (Fig. 2D). The ratio of RNA catabolic and metabolic processes was also 20%, and RNA polyadenylation was included in the pathway (Fig. 2E). Table 4 lists the GO categories and proteins found in abundance in the Responder group.

**Table 4:** List of gene ontology (GO) categories associated with proteins abundant in the Responder group

GOID	GOTerm	Associated Genes Found
GO:0032729	Positive regulation of interferon-gamma production	[FADD, HLA-DPB1]
GO:0034109	Homotypic cell–cell adhesion	[ANK3, CD99]
GO:0044291	Cell–cell contact zone	[ANK3, NECTIN2]
GO:0035456	Response to interferon-beta	[PNPT1, STING1]
GO:0035458	Cellular response to interferon-beta	[PNPT1, STING1]
GO:0061640	Cytoskeleton-dependent cytokinesis	[ANK3, CHMP7]
GO:0000281	Mitotic cytokinesis	[ANK3, CHMP7]
GO:0000959	Mitochondrial RNA metabolic process	[GRSF1, PNPT1]
GO:0043631	RNA polyadenylation	[GRSF1, PNPT1]
GO:0000957	Mitochondrial RNA catabolic process	[GRSF1, PNPT1]
GO:0000960	Regulation of mitochondrial RNA catabolic process	[GRSF1, PNPT1]
GO:0000962	Positive regulation of mitochondrial RNA catabolic process	[GRSF1, PNPT1]
GO:0045123	Cellular extravasation	[CD99, FADD]
GO:0002691	Regulation of cellular extravasation	[CD99, FADD]
GO:0002693	Positive regulation of cellular extravasation	[CD99, FADD]
GO:0072678	T cell migration	[CD99, FADD]
GO:0072683	T cell extravasation	[CD99, FADD]
GO:0031343	Positive regulation of cell killing	[FADD, NECTIN2]
GO:0001910	Regulation of leukocyte mediated cytotoxicity	[FADD, NECTIN2]
GO:0001913	T cell mediated cytotoxicity	[FADD, NECTIN2]
GO:0001912	Positive regulation of leukocyte mediated cytotoxicity	[FADD, NECTIN2]
GO:0001914	Regulation of T cell mediated cytotoxicity	[FADD, NECTIN2]
GO:0002709	Regulation of T cell mediated immunity	[FADD, NECTIN2]
GO:0001916	Positive regulation of T cell mediated cytotoxicity	[FADD, NECTIN2]
GO:0002711	Positive regulation of T cell mediated immunity	[FADD, NECTIN2]

**Fig. 3:**
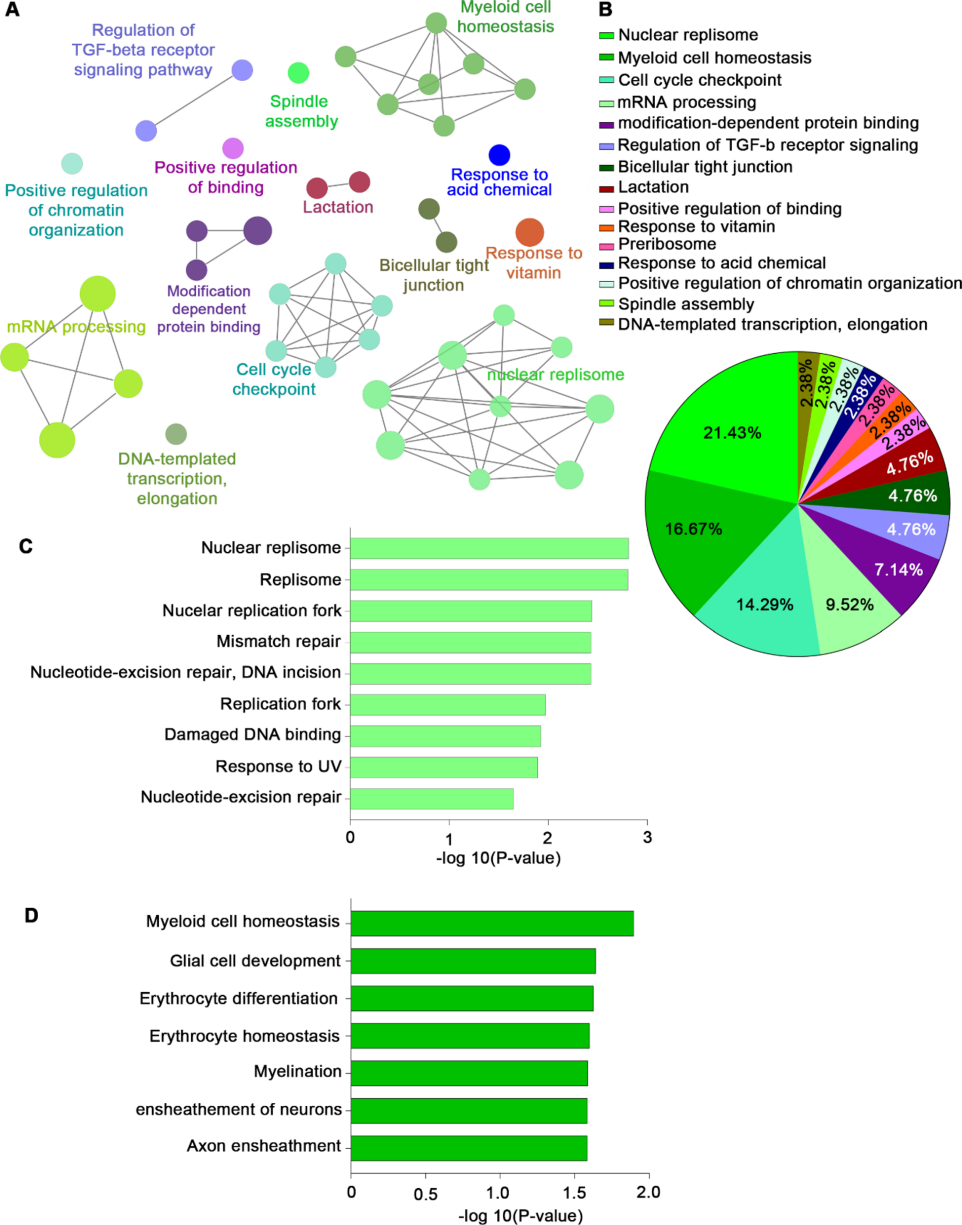
Gene ontology patterns and significant pathways in the Non-responder group. **A** Cluster analysis results in the Non-responder group. **B** Pie charts showing gene ontology classification. Bar graph of gene ontology enrichment analysis for pathways related to **C** nuclear replisome and **D** myeloid cell homeostasis

The pathways identified in the Non-responder group were commonly associated with DNA and RNA processes, both of which are essential in the nucleus. The majority of clusters were found in nucleic acid pathways, and some biomarkers were associated with pathways involved in lactation, vitamin response, and TGF-beta receptor signaling (Fig. 3A, B). The Nucleus replisome pathway, which include mismatch repair, DNA incision, and damaged DNA binding, was associated with non-responder at 21.43 percent (Fig. 3C), as well as myeloid cell homeostasis and development, and erythrocyte differentiation and homeostasis (Fig. 3D). Table 5 lists the GO categories and proteins found in abundance in the Non-responder group.

**Table 5:** List of gene ontology (GO) categories associated with proteins abundant in the Non-responder group

GOID	GOTerm	Associated Genes Found
GO:0006354	DNA-templated transcription, elongation	[ERCC2, HTATSF1, THOC5]
GO:0051225	Spindle assembly	[ARHGEF10, KIFC1, TUBGCP3]
GO:1905269	Positive regulation of chromatin organization	[GLYR1, SETDB1, SMARCB1]
GO:0001101	Response to acid chemical	[CREB1, LYN, SIPA1]
GO:0030684	Preribosome	[IGF2BP3, TSR1, WDR12]
GO:0033273	Response to vitamin	[BCHE, CCND1, SETDB1, TYMS]
GO:0051099	Positive regulation of binding	[CAV1, CLN5, ERCC2, PLCL1]
GO:0007589	Body fluid secretion	[CAV1, CCND1, CREB1]
GO:0007595	Lactation	[CAV1, CCND1, CREB1]
GO:0070160	Tight junction	[ARHGAP17, CCND1, JAM3]
GO:0005923	Bicellular tight junction	[ARHGAP17, CCND1, JAM3]
GO:1,903,844	Regulation of cellular response to transforming growth factor beta stimulus	[CAV1, LTBP4, VASN]
GO:0017015	Regulation of transforming growth factor beta receptor signaling pathway	[CAV1, LTBP4, VASN]
GO:0140030	Modification-dependent protein binding	[CBX8, GLYR1, LYN, MSH6, UBL7]
GO:0140034	Methylation-dependent protein binding	[CBX8, GLYR1, MSH6]
GO:0035064	Methylated histone binding	[CBX8, GLYR1, MSH6]
GO:0005681	Spliceosomal complex	[BCAS2, GPKOW, HTATSF1, IK, MFAP1, PRKRIP1, RBM28]
GO:0005684	U2-type spliceosomal complex	[BCAS2, HTATSF1, IK, MFAP1]
GO:0006397	mRNA processing	[BCAS2, ERCC2, GPKOW, HTATSF1, IK, MFAP1, PRKRIP1, RBM15B, RBM26, RBM28, THOC5, VIRMA]
GO:0008380	RNA splicing	[BCAS2, GPKOW, HTATSF1, IK, MFAP1, PRKRIP1, RBM15B, RBM28, THOC5, VIRMA]
GO:0000075	Cell cycle checkpoint	[CCND1, CRADD, IK, MDC1, THOC5]
GO:0007093	Mitotic cell cycle checkpoint	[CCND1, CRADD, IK, MDC1]
GO:0031570	DNA integrity checkpoint	[CCND1, CRADD, MDC1, THOC5]
GO:0000077	DNA damage checkpoint	[CCND1, CRADD, MDC1, THOC5]
GO:0044774	Mitotic DNA integrity checkpoint	[CCND1, CRADD, MDC1]
GO:0044773	Mitotic DNA damage checkpoint	[CCND1, CRADD, MDC1]
GO:0002262	Myeloid cell homeostasis	[ERCC2, JAM3, LYN, SMAP1]
GO:0007272	Ensheathment of neurons	[ARHGEF10, ERCC2, JAM3]
GO:0034101	Erythrocyte homeostasis	[ERCC2, LYN, SMAP1]
GO:0008366	Axon ensheathment	[ARHGEF10, ERCC2, JAM3]
GO:0042552	Myelination	[ARHGEF10, ERCC2, JAM3]
GO:0021782	Glial cell development	[ARHGEF10, ERCC2, LYN]
GO:0030218	Erythrocyte differentiation	[ERCC2, LYN, SMAP1]
GO:0005657	Replication fork	[BCAS2, POLD1, POLD2]
GO:0003684	Damaged DNA binding	[ERCC2, MSH6, POLD1]
GO:0009411	Response to UV	[CCND1, ERCC2, MSH6, POLD1]
GO:0043596	Nuclear replication fork	[BCAS2, POLD1, POLD2]
GO:0043601	Nuclear replisome	[BCAS2, POLD1, POLD2]
GO:0006289	Nucleotide-excision repair	[ERCC2, POLD1, POLD2]
GO:0006298	Mismatch repair	[MSH6, POLD1, POLD2]
GO:0033683	Nucleotide-excision repair, DNA incision	[ERCC2, POLD1, POLD2]

### Prognostic values of the biomarker candidates

Based on the results of LC-mass spectrometry and GO database analysis, we selected three proteins as potential biomarkers with a positive association with drug response (TMEM173, FADD, CD99) and two proteins with a potential negative association with drug response (ERCC2, POLD1) from biomarker development cohort. Among the 223 patients with recurrent glioblastoma who treated with avastin from 2017 to 2020, the 93 patients were selected for validation cohort (Table 3). For validation of the candidate biomarkers, 93 patients with high-grade GBM who recurred after surgery were selected and their TMA blocks were prepared for immunostaining.

**Fig. 4:**
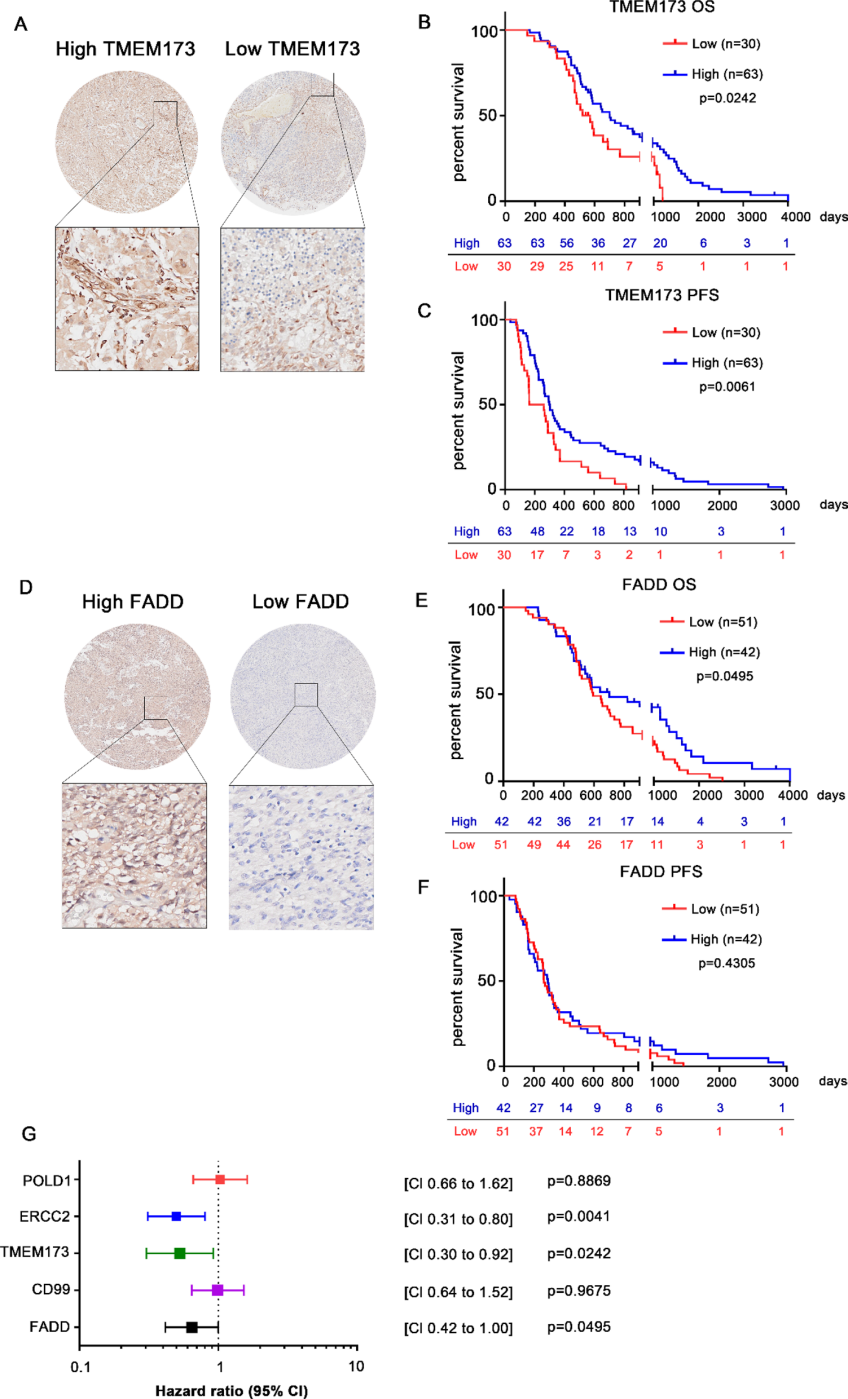
Expression of candidate biomarker proteins and survival analysis according to their expression levels. **A** Expression patterns of TMEM173 in patients with high expression levels (left) and those with low expression (right) (magnification, 20×). Log-rank analysis for **B** overall survival (OS) and **C** progression-free survival (PFS) according to the expression level of TMEM173. **D** Expression patterns of FADD in patients with high (left) or low (right) expression levels (magnification, 20 ×). Log-rank analysis of **E** OS and **F** PFS according to the expression level of FADD. **G** A Forest plot summarizing the hazard ratios for OS according to the expression level of each candidate biomarker protein

Of the 93 patients in the validation cohort, 63 patients showed high expression of TMEM173 while 30 patients showed low expression (Fig. 4A); the high expression group and the low expression group did not show significant differences in the demographic characteristics (Table 6). In terms of OS, the average of survival duration was 981 days in the high expression group and 599 days in the low expression group (P < 0.001) (Fig. 4B, Table 6). In terms of PFS, patients showed recurrence after 525 days in the high expression group and 274 days in the low expression group (P < 0.001) (Fig. 4C, Table 6).

**Table 6:** Characteristics of patients according to the expression level of TMEM173

Characteristics	High expression (n = 63)	Low expression (n = 30)	P-value
Age, years	55.1 ± 1.7	51.9 ± 2.5	0.70
Male sex	31 (49%)	13 (43%)	0.59
Pre-bevacizumab KPS score	57.8 ± 1.9	60.0 ± 2.9	0.49
Molecular type			
IDH wild type	23 (37%)	24 (80%)	0.0001
MGMT promoter status (methylated/unmethylated/NA)	16/13/34	17/6/7	
Surgical resection type			
Partial resection	21 (33%)	17 (57%)	0.03
Gross total resection	42 (67%)	13 (43%)	
Drug treatment			
Temozolomide + bevacizumab	54 (86%)	20 (67%)	
Monotherapy	9 (14%)	10 (33%)	
Temozolomide duration, days	241.7 ± 26.0	203.8 ± 30.0	0.16
Avastin dose, mg/kg	591.2 ± 15.8	591.0 ± 22.6	0.96
Treatment outcomes			
Overall survival, days	981.3 ± 100.9	599.7 ± 50.6	< 0.001
Progression-free survival, days	525.6 ± 72.4	274.7 ± 36.6	< 0.001
Initial tumor size, mm^3^	40219 ± 4196	43899 ± 7890	0.079
Recurred tumor size, mm^3^	35148 ± 4903	22780 ± 6381	0.54

Values are mean ± standard deviation or n (%), unless indicated otherwise

*KPS* Karnofsky Performance Scale; *IDH* isocitrate dehydrogenase; *NA* not available; *MGMT* O-6-methylguanine-DNA methyltransferase

In the case of FADD, 51 patients had high expression and 42 patients had low expression (Fig. 4D), and the two groups did not show significant differences in the demographic characteristics (Table 7). In the high expression group and the low expression group, the average of OS duration was 972 days and 764 days, respectively (P < 0.001) (Fig. 4E, Table 7), and the average of PFS duration was 499 days and 393 days, respectively (P < 0.001) (Fig. 4F, Table 7).

**Table 7:** Characteristics of patients according to the expression level of FADD

Characteristics	High expression (n = 42)	Low expression (n = 51)	P-value
Age, years	53.4 ± 2.0	54.5 ± 2.0	0.59
Male sex	19 (45%)	29 (57%)	0.002
Pre-bevacizumab KPS score	55.9 ± 2.3	60.85 ± 2.1	0.85
Molecular type			
IDH wild type	20 (48%)	26 (51%)	0.02
MGMT promoter status (methylated/unmethylated/NA)	17/7/18	16/12/23	
Surgical resection type			
Partial resection	19 (45%)	20 (39%)	0.56
Gross total resection	23 (55%)	31 (61%)	
Drug treatment			
Temozolomide + bevacizumab	32 (76%)	32 (76%) 42 (82%)	
Monotherapy	10 (24%)	9 (18%)	
Temozolomide duration, days	250.2 ± 35.2	216.1 ± 24.1	0.19
.Avastin dose, mg/kg	594.1 ± 16.0	588.5 ± 19.8	0.074
Treatment outcomes			
Overall survival, days	972.5 ± 136.2	764 ± 68.56	< 0.001
Progression-free survival, days	499.9 ± 100.1	393.6 ± 46.6	< 0.001
Initial tumor size, mm^3^	41753 ± 6764	41138 ± 4151	0.008
Recurred tumor size, mm^3^	30212 ± 5443	31958 ± 5661	0.41

Values are mean ± standard deviation or n (%), unless indicated otherwise

*KPS* Karnofsky Performance Scale; *IDH* isocitrate dehydrogenase; *NA* not available; *MGMT* O-6-methylguanine-DNA methyltransferase

In the case of CD99, 47 patients had high expression and 46 patients had low expression (Additional file 1: Fig. S1A). In the high expression group and the low expression group, the average of OS duration was 879 days and 836 days, respectively (P = 0.77) (Additional file 1: Fig. S1B, Table 8), and the average of PFS duration was 459 days and 426 days, respectively (P = 0.75) (Additional file 1: Fig. S1C, Table 8).

**Table 8:** Characteristics of patients according to the expression level of CD99

Characteristics	High expression (n = 47	Low expression (n = 46)	P-value
Age, years	54.6 ± 1.9	53.4 ± 2.0	0.68
Male sex	30	20	< 0.05
Pre-bevacizumab KPS score	60.7 ± 2.1	56.3 ± 2.4	0.16
Molecular type			
IDH wild type	43	41	0.7
MGMT promoter status (methylated/unmethylated/NA)	11/6/30	23/8/16	
Surgical resection type			
Partial resection	18	20	0.67
Gross total resection	29	27	
Drug treatment			
Temozolomide + bevacizumab	19	41	< 0.05
Monotherapy	28	5	
Temozolomide duration, days	260.7 ± 31.0	191.5 ± 22.2	0.09
.Avastin dose, mg/kg	605.1 ± 15.5	576.5 ± 20.7	0.27
Treatment outcomes			
Overall survival, days	879 ± 99.6	836.9 ± 106.4	0.77
Progression-free survival, days	459.1 ± 74.7	426.3 ± 72.5	0.75
Initial tumor size, mm^3^	42035 ± 5857	40775 ± 4863	0.87
Recurred tumor size, mm^3^	31187 ± 5880	31137 ± 5298	0.99

We expected that high expression levels of ERCC2 and POLD1 would be negatively associated with survival outcomes. In the case of ERCC2, 48 patients had high expression and 45 patients had low expression (Additional file 1: Fig. S2A). In the high expression group and the low expression group, the average of OS duration was 1082 days and 619 days, respectively (P = 0.001) (Additional file 1: Fig. S2B, Table 9), and the average of PFS duration was 588 days and 289 days, respectively (P = 0.003) (Additional file 1: Fig. S2C, Table 9). Contrary to our expectation, expression of ERCC2 had a positive correlation with survival outcomes.

**Table 9:** Characteristics of patients according to the expression level of ERCC2

Characteristics	High expression (n = 48	Low expression (n = 45)	P-value
Age, years	53.15 ± 1.915	54.91 ± 2.049	0.53
Male sex	25	23	0.93
Pre-bevacizumab KPS score	60.68 ± 2.262	56.36 ± 2.207	0.18
Molecular type			
IDH wild type	34	43	< 0.05
MGMT promoter status (methylated/unmethylated/NA)	13/3/22	20/8/17	
Surgical resection type			
Partial resection	21	17	0.56
Gross total resection	27	28	
Drug treatment			
Temozolomide + bevacizumab	29	29	0.68
Monotherapy	19	16	
Temozolomide duration, days	268 ± 30.71	176.1 ± 18.51	0.03
.Avastin dose, mg/kg	610.7 ± 18.31	571.6 ± 17.44	0.13
Treatment outcomes			
Overall survival, days	1082 ± 121.4	619.2 ± 58.07	0.001
Progression-free survival, days	588.2 ± 90.81	289.4 ± 33.99	0.003
Initial tumor size, mm^3^	43443 ± 5978	39304 ± 4679	0.59
Recurred tumor size, mm^3^	28949 ± 5759	33476 ± 5389	0.57

Lastly, in the case of POLD1, 58 patients had high expression and 51 patients had low expression (Additional file 1: Fig. S3A). In the high expression group and the low expression group, the median OS was 878 days and 824 days, respectively (P = 0.72) (Additional file 1: Fig. S3B, Table 10), and the average of PFS duration was 424 days and 471 days, respectively (P = 0.66) (Additional file 1: Fig. S3C, Table 10). According to our expectation, POLD1 was negatively associated with survival outcomes, albeit without statistical significance.

**Table 10:** Characteristics of patients according to the expression level of POLD1

Characteristics	High expression (n = 58)	Low expression (n = 35)	P-value
Age, years	52.37 ± 2.385	54.98 ± 1.716	0.37
Male sex	30	18	0.98
Pre-bevacizumab KPS score	58.11 ± 1.981	59.14 ± 2.669	0.75
Molecular type			
IDH wild type	50	28	0.43
MGMT promoter status (methylated/unmethylated/NA)	16/5/37	17/6/9	
Surgical resection type			
Partial resection	19	18	0.07
Gross total resection	39	17	
Drug treatment			
Temozolomide + bevacizumab	40	22	0.54
Monotherapy	18	13	
Temozolomide duration, days	243.6 ± 29.89	209.1 ± 21.91	0.41
.Avastin dose, mg/kg	593 ± 15.84	588.3 ± 21.95	0.86
Treatment outcomes			
Overall survival, days	878.6 ± 96.25	824.4 ± 109.4	0.72
Progression-free survival, days	424.2 ± 61.62	471.2 ± 93.68	0.66
Initial tumor size, mm^3^	44909 ± 5323	35735 ± 4914	0.24
Recurred tumor size, mm^3^	33590 ± 5377	26970 ± 5404	0.42

Figure 4G shows the hazard ratios (HRs) and their 95% confidence intervals (CIs) of each biomarker candidate for overall survival. High expressions of TMEM173 (HR, 0.53; 95% CI 0.30–0.92; P = 0.024), FADD (HR, 0.65; 95% CI 0.42–1.00; P = 0.0495), and ERCC2 (HR, 0.50; 95% CI 0.31–0.80; P = 0.004) were significantly associated with better overall survival in patients with recurrent GBM.

## Discussion

Development of novel biomarkers that can accurately predict the response to anti-angiogenic treatment in patients with recurrent glioblastoma is of crucial importance. Additionally, potent biomarkers can predict the adverse effects of anticancer drugs, which can lead to potential cost savings [42, 43]. Currently, a variety of indicators are used to assess the response to anti-angiogenic therapies in recurrent glioblastoma, including non-invasive diagnostic biomarkers such as phosphatidylinositol-glycan biosynthesis class F (PIGF) [44], interleukin-8 (IL-8) [45] and circulating collagen IV [46]. Furthermore, K^trans^ MR imaging techniques can also be used to assess a patient's response to treatment in cases of recurrent glioblastoma [44]. After surgical treatment, CD31 staining in tumor tissues can be used to determine the micro-vessel density, which is not associated with drug reactivity but was identified in tumor tissues via CA9 (Carbonic Anhydrase 9), a hypoxia marker that was overexpressed in patients with a short-term survival [47]. Additionally, as a predictor of non-reactivity, elevations in SDF-1 alpha levels are found in patients with recurrent glioblastoma showing tumor progression, and elevations in TIE2 (TEK receptor tyrosine kinase 2) are also observed in association with tumor progression [45]. Circulating biomarkers such as those in the plasma and PBMCs are more easily and rapidly detectable than those in solid tumors, which require surgical assessment.

When validated, TMEM173, which was frequently detected in patients with a response to anti-angiogenic treatment, demonstrated a pattern of surviving an additional 381.6 days on average. TMEM173, which recognizes cancer cell DNA fragments, is expressed at a high level in endothelial cells that can infiltrate immune cells into tumor sites and normalize the surrounding blood vessels [48]. While TMEM173 cannot directly bind to VEGF receptors, it could contribute to the transformation of non-inflamed tumors into inflamed tumors. Patients with elevated levels of TMEM173 may particularly benefit from combination therapy with anti-angiogenic therapy. Considering the tumor resistance against anti-angiogenic therapy is associated with low-level immune reaction, TMEM173 that could enhance immune response via tumor vessel normalization.

FADD is involved in necroptosis, which aids in both tumor formation and suppression [49]. Inhibition of tumor formation is accomplished by the priming of anti-tumor CD8 + T cells via DMAP signaling [50]. According to the gene ontology analysis in this study, FADD can initiate anti-tumor necroptosis and aid the process of T cell-mediated cytotoxicity. In terms of overall survival, the average survival period was 647 days in patients with low FADD expression and 900 days in patients with high FADD expression.

CD99, which is an o-glycosylated transmembrane protein, was identified as a response-related marker involved in T cell migration and extravasation process in our gene ontology analysis. CD99 is also used as a diagnostic marker for Ewing's sarcoma and is involved in tumor cell migration. CD99 in tumor blood vessels inhibits tumor formation [51]; however, CD99 expression is increased in glioblastoma patients, and when divided according to molecular type, CD99 expression is higher in the mesenchymal type than in the pro-neural type. Additionally, an in vitro study using U87 MG showed that when CD99 was suppressed, tumor cell migration was decreased [52]. In our study cohort, the ratio of high- to low-expression patients was approximately 1:1, and there was no significant difference in the survival or recurrence rates according to the degree of CD99 expression. Due to the lack of molecular type analysis in this study, statistical significance might not be verified. However, we suggest that comparing CD99 expression in GBM patients with mesenchymal type may be useful in demonstrating drug reactivity.

As a non-response prediction biomarker found in this study, ERCC2 is a component of the nuclear excision repair process that recovers DNA damaged by environmental mutations such as radiation and ultraviolet light. [53, 54]. As ERCC2 was highly expressed in the non-responder group, we expected that lower expression of ERCC2 in the validation cohort would be associated with a better survival rate; however, the survival analysis showed an opposite result in which patients with high expression of ERCC2 had a significantly higher OS. This unexpected result may be at least partially due to the fact that polymorphism cannot be detected by immunostaining. Therefore, the expression of ERCC2 should be evaluated at the gene level.

POLD1 is a nuclear replication enzyme and although it was highly expressed in non-responders, its expression level was not associated with significant differences in survival or recurrence in our study. POLD1 has been studied in hereditary colon cancer and endometrial cancer [55, 56], but it has yet to be investigated in glioblastoma. POLD1 appears to be a biomarker for predicting prognosis in cases of hereditary cancer.

Among the five molecules found in this experiment (TMEM173, FADD, CD99, ERCC2, and POLD1), TMEM173 and FADD may be considered as potential biomarkers that can assist the treatment of patients using anti-angiogenic therapy. The expression of other three biomarkers was related to DNA damage; however, as all tumor cells have some degree of DNA damage, their potential usefulness in GBM should be evaluated using different experimental approaches.

## Conclusion

By performing a comprehensive proteomic analysis in GBM patients with recurrence, we found that TMEM173 and FADD may be used to predict the response to anti-angiogenic therapy and prognosis before recurrence. Evaluating the expression of these biomarkers may be helpful in determining the treatment regimen of patients with glioblastoma.

## Supplementary Information

** Additional file 1: Fig. S1.** Expression of CD99 and survival analysis according to its expression levels. **A,** Expression patterns of CD99 in patients with high expression levels (left) and those with low expression levels (right) (magnification, 20×). Log-rank analysis for **B,** overall survival (OS) and **C,** progression-free survival (PFS) according to the expression level of CD99. **Fig. S2.** Expression of ERCC2 and survival analysis according to its expression levels. **A**, Expression patterns of ERCC2 in patients with high expression levels (left) and those with low expression levels (right) (magnification, 20×). Log-rank analysis for **B,** overall survival (OS) and **C,** progression-free survival (PFS) according to the expression level of ERCC2. **Fig. S3.** Expression of POLD1 and survival analysis according to its expression levels. **A**, Expression patterns of POLD1 in patients with high expression levels (left) and those with low expression levels (right) (magnification, 20×). Log-rank analysis for **B**, overall survival (OS) and **C**, progression-free survival (PFS) according to the expression level of POLD1.

## Data Availability

All data generated or analyzed during this study are included in this published article and its Additional information files. Further information is available from the corresponding author (rghree@amc.seoul.kr) upon request.

## Abbreviations

ATRX: Alpha-thalassemia/mental retardation, X-linked
CA9: Carbonic Anhydrase 9
CCRT: Concurrent chemoradiation therapy
CD99: Cluster of differentiation 99
CEACAM1: Carcinoembryonic antigen-related cell adhesion molecule 1
CT: Computed tomography
EGFR: Epidermal growth factor receptor
ERCC2: Excision repair cross-complementation group 2
FADD: Fas associated via death domain
FDR: False discovery rate
FGF2: Fibroblast growth factor 2
FPR1: Formyl peptide receptor 1
GO: Gene-ontology
IL-8: Interleukin-8
LC-HRMS: Liquid chromatography-high resolution mass spectrometry
MAPK: Mitogen-activated protein kinase
MCT1: Monocarboxylate transporter 1
MGMT: O-6-methylguanine-DNA methyltransferase
MMP9: Matrix metalloproteinase 9
MRI: Magnetic resonance imaging
NER: Nuclear excision repair
OS: Overall survival
PCA: Principle component analysis
PDGF: Platelet-derived growth factor
PFS: Progression-free survival
PIGF: Phosphatidylinositol-glycan biosynthesis class F
POLD1: DNA polymerase delta 1, catalytic subunit
S100A8: S100 calcium binding protein A8
SOCS3: Suppressor of cytokine signaling 3
TERT: Telomerase reverse transcriptase
TIE2: TEK receptor tyrosine kinase 2
TMA: Tissue microarray
TMEM173: Transmembrane protein 173
TNF-alpha: Tumor necrosis factor-alpha
VEGF: Vascular endothelial growth factor
