# Supplementary material for: Proteomic analysis predicts anti-angiogenic resistance in recurred glioblastoma
Source: J Transl Med. 2023 Feb 2;21:69. doi: 10.1186/s12967-023-03936-8 (PMC9893563; doi:10.1186/s12967-023-03936-8)
Supplement: Supplementary file 1 — Additional file 1: Fig. S1. Expression of CD99 and survival analysis according to its expression levels. A, Expression patterns of CD99 in patients with high expression levels (left) and those with low expression levels (right) (magnification, 20×). Log-rank analysis for B, overall survival (OS) and C, progression-free survival (PFS) according to the expression level of CD99. Fig. S2. Expression of ERCC2 and survival analysis according to its expression levels. A, Expression patterns of ERCC2 in patients with high expression levels (left) and those with low expression levels (right) (magnification, 20×). Log-rank analysis for B, overall survival (OS) and C, progression-free survival (PFS) according to the expression level of ERCC2. Fig. S3. Expression of POLD1 and survival analysis according to its expression levels. A, Expression patterns of POLD1 in patients with high expression levels (left) and those with low expression levels (right) (magnification, 20×). Log-rank analysis for B, overall survival (OS) and C, progression-free survival (PFS) according to the expression level of POLD1. [file 12967_2023_3936_MOESM1_ESM.docx]

**Fig. S1**


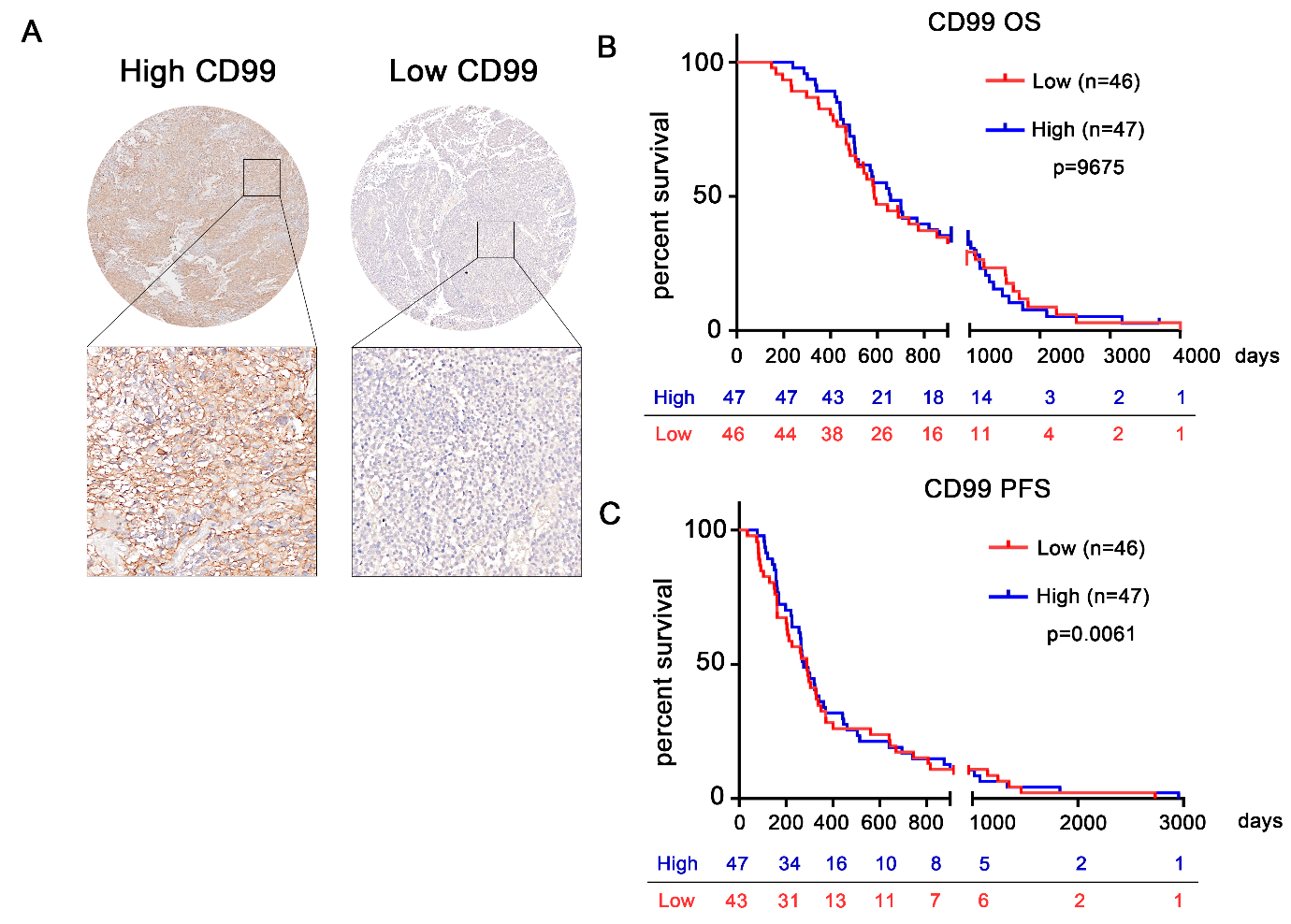


**Fig. S2**

**
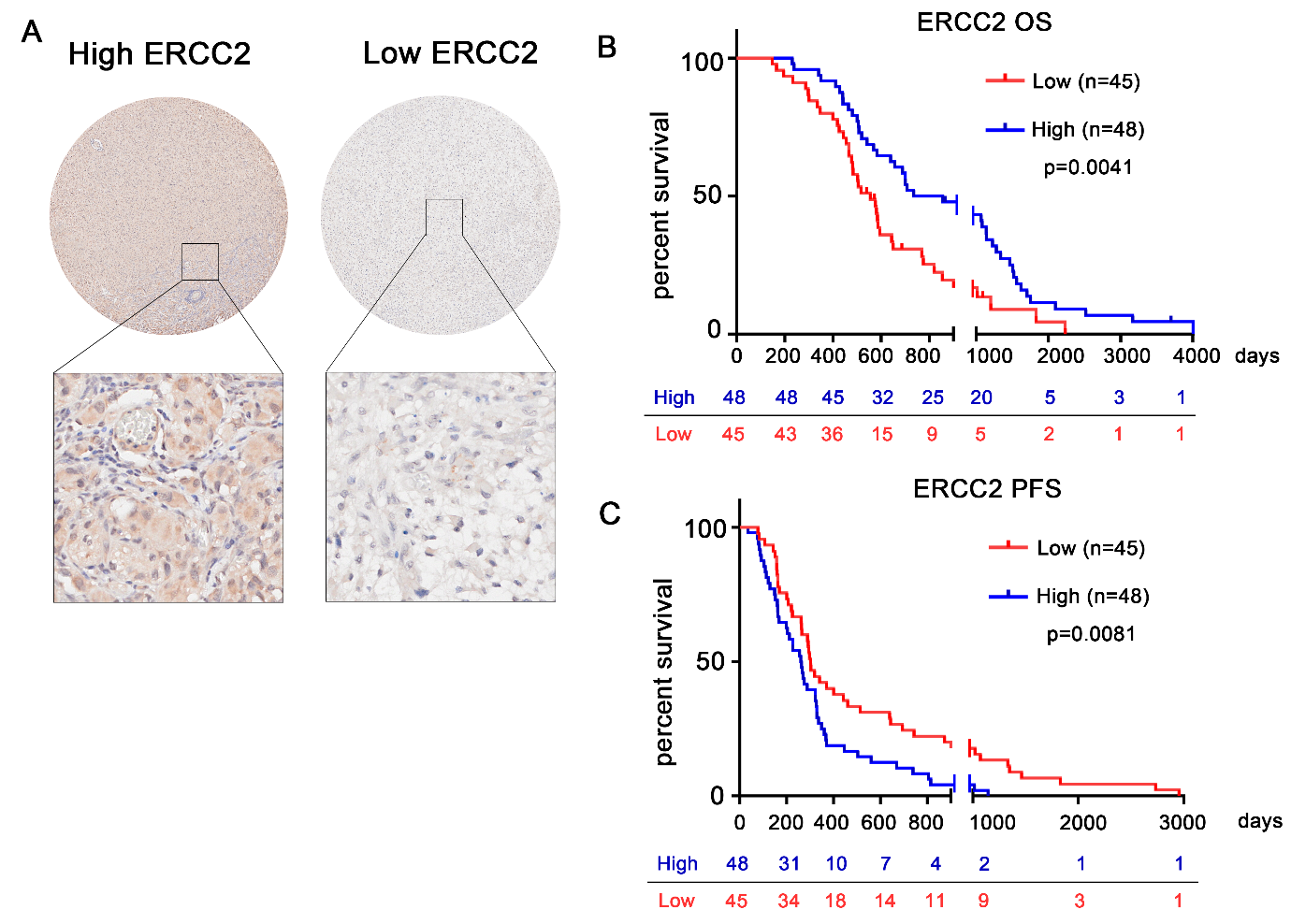
**

**Fig. S3**


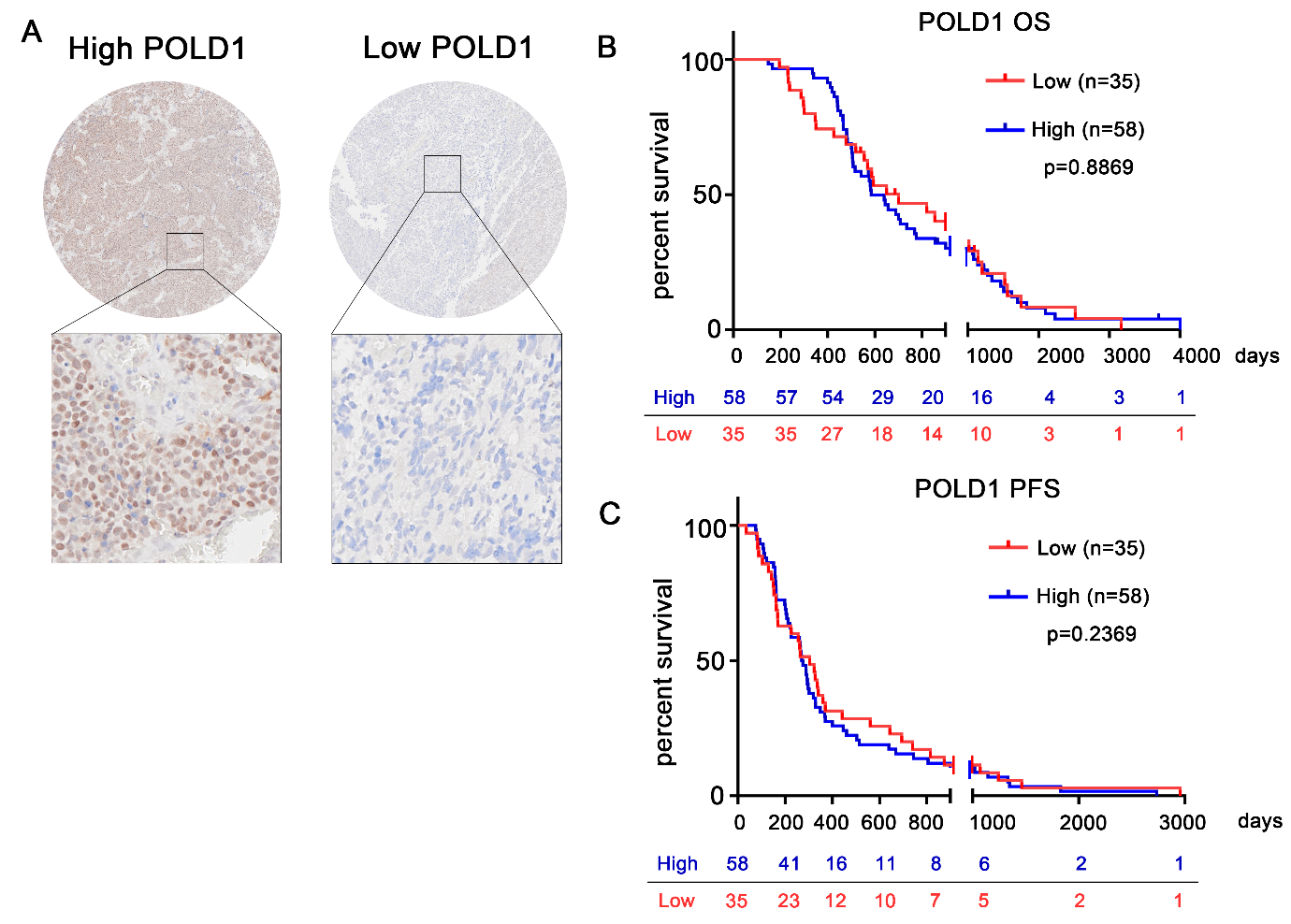


**Fig. S1. Expression of CD99 and survival analysis according to its expression levels.**

**A,** Expression patterns of CD99 in patients with high expression levels (left) and those with low expression levels (right) (magnification, 20×). Log-rank analysis for **B,** overall survival (OS) and **C,** progression-free survival (PFS) according to the expression level of CD99.

**Fig. S2. Expression of ERCC2 and survival analysis according to its expression levels.**

**A**, Expression patterns of ERCC2 in patients with high expression levels (left) and those with low expression levels (right) (magnification, 20×). Log-rank analysis for **B,** overall survival (OS) and **C,** progression-free survival (PFS) according to the expression level of ERCC2.

**Fig. S3. Expression of POLD1 and survival analysis according to its expression levels.**

**A**, Expression patterns of POLD1 in patients with high expression levels (left) and those with low expression levels (right) (magnification, 20×). Log-rank analysis for **B,** overall survival (OS) and **C,** progression-free survival (PFS) according to the expression level of POLD1.
